# Communal breeding promotes a matrilineal social system where husband and wife live apart

**DOI:** 10.1098/rspb.2013.0010

**Published:** 2013-05-07

**Authors:** Jia-Jia Wu, Qiao-Qiao He, Ling-Ling Deng, Shi-Chang Wang, Ruth Mace, Ting Ji, Yi Tao

**Affiliations:** 1 Key Laboratory of Animal Ecology and Conservation Biology, Centre for Computational Biology and Evolution, Institute of Zoology, Chinese Academy of Sciences, Beijing 100101, People's Republic of China; 2 Key State Laboratory of Vegetation and Environmental Change, Institute of Botany, Chinese Academy of Sciences, Beijing, People's Republic of China; 3 Department of Anthropology, University College London, 14 Taviton Street, London WC1H 0BW, UK

**Keywords:** anthropology, kinship, matrilineal

## Abstract

The matrilineal Mosuo of southwest China live in large communal houses where brothers and sisters of three generations live together, and adult males walk to visit their wives only at night; hence males do not reside with their own offspring. This duolocal residence with ‘walking’ or ‘visiting’ marriage is described in only a handful of matrilineal peasant societies. Benefits to women of living with matrilineal kin, who cooperate with child-care, are clear. But why any kinship system can evolve where males invest more in their sister's offspring than their own is a puzzle for evolutionary anthropologists. Here, we present a new hypothesis for a matrilineal bias in male investment. We argue that, when household resources are communal, relatedness to the whole household matters more than relatedness to individual offspring. We use an inclusive fitness model to show that the more sisters (and other closely related females) co-reside, the more effort males should spend working on their sister's farm and less on their wife's farm. The model shows that paternity uncertainty may be a cause of lower overall work rates in males, but it is not likely to be the cause of a matrilineal bias. The bias in work effort towards working on their natal farm, and thus the duolocal residence and ‘visiting marriage’ system, can be understood as maximizing inclusive fitness in circumstances where female kin breed communally.

## 1. Introduction

Human families are very diverse, showing a wide range of residence patterns, be it among hunter–gatherers, farmers or others; among farmers, females usually but not always disperse at marriage [1]. A significant minority of human societies have matrilocal residence, in which males disperse [2]. Societies with matrilocal residence are generally associated with a suite of other matrilineal biases in descent, and inheritance, where a male transmits property and titles to his sister's sons rather than his own sons; and the ownership of the natal home and land are normally passed from mother to daughter. There is a range of evidence to suggest that women benefit from the proximity of matrilineal kin, especially with help raising offspring [3,4], which has lead some anthropologists to describe humans as communal breeders [5–7]. However it is not clear why males tolerate a system that favours investment in their sister's rather than their own offspring [8]. Inclusive fitness models do suggest matrilineal inheritance can evolve under very high levels of paternity uncertainty [9,10]; or that matrilineal social organization is only likely to be an evolutionary stable strategy when males are polygynous [11]. Here, we suggest a new hypothesis for a matrilineal bias in male investment, which is that working for sisters can evolve when female kin breed communally.

Patrilineal inheritance is associated with polygyny and male-biased wealth inheritance [12], whereas matrilineal systems are more often associated with a lack of high value, controllable resources [13,14]. Paternity certainty is usually strongly controlled in patrilineal systems, so that males can ensure that the inheriting sons are closely related to them. By contrast, in most matrilineal societies, marriage bonds are usually weak and paternity uncertainty is thought to be high [9]. In most matrilineal systems, males disperse and reside uxorilocally, where they are expected to work on their wife's family farm, but in a few societies neither sex disperse (known as duolocal or natalocal residence). This is the case of the Mosuo of southwestern China (and a very small number of other Asian matrilineal peasant societies). Here not only daughters but also sons stay in their natal home throughout life. Mosuo can marry, but as males are not co-resident with their spouse and offspring, visiting their wives only at night, the marriage system is often described as ‘walking’ or ‘visiting marriage’.

**Figure 1.**
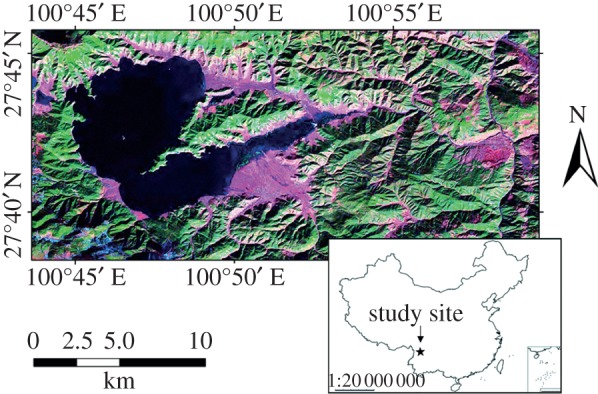
Regional satellite map of the study area. The Mosuo inhabit strips of farmland around the shores of Lugu Lake in Sichuan and Yunnan Provinces, Southwestern China (downloaded from https://zulu.ssc.nasa.gov/mrsid/mrsid.pl, by Applied Science and Technology Project Office, John C. Stennis Space Center). The star shows the location of Lugu Lake within China.

The Mosuo (also known as the Na) inhabit strips of farmland near and around the shores of Lugu Lake in southwestern China, which covers a geographically constrained habitat surrounded by steep and forested hills that are not suitable for farming (figure 1). Diets used to be supplemented by fishing and hunting, but wildlife resources are now depleted. The group is related to Tibetans and Naxi [15] and speaks a Tibeto-Burman language [16]. Land is farmed by matrilineal family groups, although is technically on lease from the Chinese government, and prior to the revolution much of it was under the ultimate control of an aristocracy [17]. Mosuo families live in large matrilineal households of three generations of brothers and sisters and the matrilineal offspring. Thus, there are usually several co-resident breeding women; they are breeding communally in the sense that they cooperate with child-care, domestic and farm labour, and share all the household resources [16]. The wives and children of the males reside elsewhere with their matrilineal kin.

Mosuo houses are large structures, traditionally built around a courtyard. The grandmother is usually head of the household, and the house is centred around a large grandmother's room, where the children also sleep and guests are received at a central fireplace [18]. The grandmother plays a key role in running the household, providing a large portion of the child-care as well as continuing to help with farming and feeding the family. Sisters and adult daughters have their own rooms, where their husbands visit them at night; husbands do not eat in their wife's household (except in rare circumstances, for example, if the husband is helping the family with his labour during the planting season) [17]. Males eat in their natal household but are expected either to visit their wives at night, or share another room in the house with the unmarried men and boys. Senior men may get their own room in larger houses. Mosuo females work hard cooperating in both the domestic arena, including child-care and cooking as well as doing the majority of the agricultural labour; men help with agricultural labour at planting and harvest but are rarely seen in the fields at other times [19]. Men do more market trading (sometimes long-distance trading) and building [20]. There are historical accounts of men spending a large amount of time in monasteries; and some had to work on the land of aristocrats as serfs or to pay off debts [18]. Husbands are expected to help on their wives farms if asked, but agricultural labour is highly communal during periods of high labour demand, when most people help on their relatives farms, and on neighbours' farms, to some extent [20]. Households normally feed all those who help with work on their farm that day.

Mosuo marriage is not marked by a very elaborate ceremony, if any, but the survival of a child to one month, especially the first child, is now marked by the father's family acknowledging the birth with gifts [20]. Divorce is assumed to have occurred if a husband has stopped visiting for some time and remarriage can then occur.

Matrilineal inheritance is a puzzle for both social and evolutionary anthropologists, as males are normally assumed to be dominant and a system where they invest more in sisters offspring than their own is hard to reconcile with maximizing inclusive fitness [21]. Inclusive fitness models of matriliny have suggested that matrilineal investment is favoured if paternity certainty is lower than 0.268 [9]. It has been pointed out that this figure might be misleading as it is the number of siblings that share fathers that is important [22]. However, estimates of paternity uncertainty in human societies do not appear to be anywhere near high enough for this to be what is maintaining matrilineal social organization (one of the highest estimates being in the Himba who report only 17% extra-pair paternity [23]). Another model shows that matrilineal social organization is likely to arise only when males are polygynous, and when returns on resources conform to certain functions characteristic of more extensive systems [11]. Data on genetic paternity are not available for the Mosuo. High levels of promiscuity are described in some ethnographic accounts of the Mosuo from the recent past [18–20], but that did not match our own observations on reported number of fathers per woman now (see §4). The Chinese government favours births within monogamous marriage, and since 1980s, there have been restrictions on having more than three children in this region, both of which may have reduced the reporting and incidence of children of one mother having different fathers.

Here, we develop an inclusive fitness model of the optimal allocation of male effort on his wife's or his sister's farm, which investigates how his investment depends both on *p* (paternity certainty) and also the number of female kin that are breeding communally. We collected data from a large population of Mosuo in Sichuan Province, China, on relatedness, working patterns on farms and age at first birth to test both the assumptions and predictions of the model. Our results are consistent with the model prediction that, whether or not females or males are mating polygamously, if female kin are breeding communally, then males can maximize inclusive fitness by working predominantly for their natal household, which is the household to which they are most related.

## 2. An inclusive fitness model of optimal male allocation of effort

We model the case where a male has to decide how to allocate his investment between his wife's household and his natal household, in order to maximize inclusive fitness [21]. The full details of the model are described in the electronic supplementary material. We assume that investment on a farm generates a quantity of food (or other benefit), rising towards an asymptote as the amount of investment on a particular household increases; this is because we assume the total amount of work any one person can achieve is limited, as the size of a harvest increases its marginal benefit to the nutritional state of the household diminishes and once all the fields a household owns are planted or harvested, additional labour is less useful. We assume that all food generated is shared by all household residents, in line with our own observation and that of other enthographic accounts [18,19]. The inclusive fitness benefit of that effort thus depends on how related the male is to offspring produced by all the breeding females in that household. We assume the *p* (paternity certainty) is determined by females. We use the model to explore how both *p* and the number of sisters (or other closely related female kin) co-residing in households influences male allocation of effort. We also model how allocation changes when we include time allocation to activities that increase male attractiveness in addition to working on either sisters' or spouses' farms; this activity is assumed to have a fitness benefit directly related to *p*.

**Figure 2.**
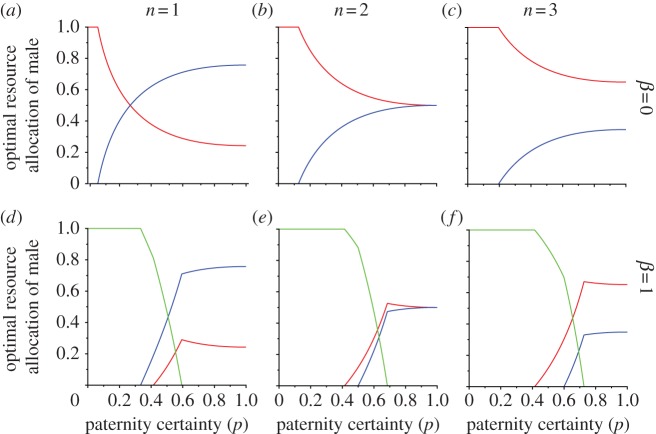
The effect of paternity certainty (*p*) on optimal male allocation of relative effort to either his wife's farm (blue line, y*) or his sister's farm (red line, x*) when *x* + *y* = 1; (*a*) when households include one breeding female only; (*b*) when households include two sisters breeding communally; (*c*) when households include three sisters breeding communally. It is assumed *p* is controlled by females. (*d*–*f*) The effect of paternity certainty (*p*) on optimal male relative allocation of effort to his wife's farm (blue line, y*) or his sister's farm (red line, x*) or to activities that promote extra-pair reproductive success (green line, 1−x*−y*); (*d*) when there is only one breeding female per household; (*e*) when households include two sisters breeding communally and (*f*) when households include three sisters breeding communally.

Figure 2*a*–*c* shows the optimal division of male effort between working for his wife's family farm (y*) or on his natal family farm (x*), when *x* + *y* = 1; figure 2*d*–*f* and electronic supplementary material, figure S1 shows the optimal division of male effort between working for his wife's family (y*), on his natal family farm (x*), or working on neither farm (1−x*−y*), when *x*+*y* < 1. We show scenarios where women either breed singly (figure 2*a*,*d*) or breed communally with either one (figure 2*b*,*e*) or two (figure 2*c*,*f*) sisters. In the electronic supplementary material, figure S2, we also show model scenarios in which communal households include some female cousins. The optimal allocation of effort to each farm is shown according to *p*. It is clear that the more sisters (or other closely related female kin) breed communally, the relatively more effort males allocate to their natal family farm.

If households include only one breeding female, it is best for males to spend the majority of their effort working on their wife's farm relative to their sister's farm; so if this case were the norm then it is likely that males would reside with their wife. In agreement with Greene [9], our model also predicts that only if *p* < 0.268 is working on your sister's farm favoured over working on your wife's farm, if households include only one breeding female [9]. This is a conservative method of calculating relatedness with sister's kin, as Rogers has pointed out [22], and if we used other measures that took into account that ‘extra-pair’ fathers may father more than one child per family, then there would be more of a matrilineal bias in investment at a higher threshold of paternity certainty. However, the optimal relative allocation does include spending a minority of time working on their sister's farm, even when the male is less related to his sister's offspring than his wife's offspring, owing to the asymptotic nature of the assumed benefits of help on any one farm (figure 2*a*).

If sisters generally breed communally, then the situation changes; the optimal strategy for males is to spend relatively more effort on their sisters' farm, as the fitness benefits of investing in their wife's farm are diluted by helping to feed unrelated children of the wife's sisters or other kin. The larger the communal households, the more pronounced this effect becomes, favouring males to work predominantly on their natal family farm if breeding females per farm is two or more. This would presumably favour duolocal residence, as it is unlikely men would be fed in their wife's household if most of their work effort was for the benefit of another household (figure 2*b,c*). The effect of increasing paternity uncertainty is relatively small under a realistic range of values. When *p* is high or even moderately high (*p* well above 0.5), while there is an increasing allocation of effort to your sisters' farm relative to your wife's farm as paternity uncertainty increases, it is a small effect compared with the effects of sororal communal breeding.

We develop the model to allow males to allocate effort to any behaviour that will increase attractiveness, and thus extra-pair mating success, which does not involve working on your wife's or sister's farm. Behaviour that enhances mating success could include activities such as engaging in social activities, preserving energy by eating or resting, or engaging in group activities, such as politics or warfare, that enhance prestige. As paternity certainty decreases, effort allocated to this activity becomes relatively more important than farm work; and if paternity certainty gets very low, then the main effect is that male allocation of effort to either farm reduces markedly or stops completely, rather than for relative male investment in sisters to increase (figure 2*d*–*f*). Thus, it appears that high levels of paternity uncertainty would be more likely to reduce male effort spent on any farming rather than favouring matrilineal biases in effort.

The fitness benefits to a male for enhancing his attractiveness to extra-pair females are not known; but we assume the benefits of mating effort are linear, as extra-pair matings are clandestine and do not involve labouring on farms, and the fitness resulting from such behaviour is more likely to be a function simply of the number of females attracted, which is not necessarily limited. In the electronic supplementary material, we show that the magnitude of the benefit of these activities has a quantitative rather than a qualitative effect on the results (see the electronic supplementary material, figures S1 and S2). Overall our model suggests that it is not paternity uncertainty but communal breeding among sisters that is generating duolocal residence and the visiting marriage.

## 3. Data and methods

In 2007, we conducted a demographic census of 7034 people in five Mosuo villages in Lugu Lake Town on the shores of Lugu Lake in the Tibetan borderlands of Sichuan province, China. Lugu Lake Town is an area of about 283 km^2^, and the total population is about 10 000. Most of the inhabitants are Mosuo, and others are Yi, Han, Pumi and Tibetan people. For each household, one adult representative was interviewed about the personal information of all male and female family members as well as household information, which included name, ethnic group, gender, year of birth, animal sign of the year of birth, education level, parents' name, marriage status, type of marriage residence, spouse's name, children's name, children's year of birth, children's gender, and address of residence, global positioning system location of residence, land size, number of livestock, number of poultry, number of hotels and businesses. Age at first birth of each men and women were calculated from the year of birth of the first child.

Relatedness between each pair of Mosuo individuals was calculated using Descent (v. 0.2, copyright 2003–2005, Edward H. Hagen) based on genealogical data; average relatedness of Mosuo individuals to males, females, and all members in the natal household and in the spouse's household were also calculated.

In the planting season of 2011 and 2012, we also conducted spot observations on who was working on the land belonging to a random sample of farms, recording all the personal information of each individual seen working on a field, including name, gender, ethnic group, age, animal sign of the year of birth, and relationship with the owner of the land, taken from a random sample of 159 farms in three villages. Based on relationships with the owner of the land, we defined whether each individual was the owner himself or herself, or was helping matrilineal kin, patrilineal kin, spouse, neighbours or others.

Two-way ANOVA analyses were carried out using R software (v. 2.15.1) and all figures were done using IBM SPSS (v. 18.0, SPSS Inc.). Models and graphics from models use Maple v. 15 software and full details of the modelling procedures are given in the electronic supplementary material.

## 4. Results

### (a) Residence patterns and reproduction

We found duolocal residence was still the most common form of marital residence among the Mosuo at our study site in Lugu Lake (55% of adult males and 62% of adult females in our sample of 1059 males and 1411 females). Each duolocal household contained a mean of 2.14 (range 0–7, s.d. = 1.33, *n* = 210 duolocal households) breeding-age females per household. Less than 5 per cent of visiting marriage mothers sent children to live with their fathers. In a sub-sample of households, we had data on the geographical distance between husband and wife's household and 70 per cent of duolocal males live less than 5 km from their wives' household (*n* = 495 males). Males rarely bring their wives to live with them in their natal household (only 6% of females live virilocally), and females also rarely bring their husbands to live in their household (5% males live uxorilocally). These tended to be temporary strategies to overcome an imbalance of sexes in a household and the family usually reverted to duolocal residence in the next generation.

**Figure 3.**
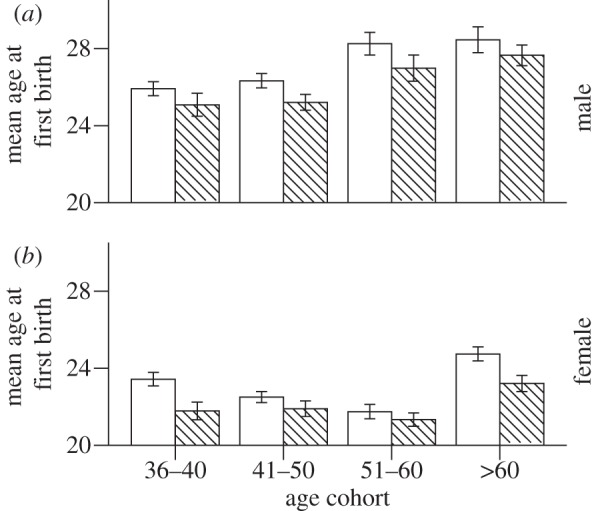
Effects of age cohort and marriage residence upon age at first birth of fertile Mosuo (*a*) males (*n* = 620) and (*b*) females (*n* = 831). Error bars indicate mean±s.e. (*a*) Mean age of first birth of males was significantly different among age cohort (two-way ANOVA, *F*_3,612_ = 11.535, *p* < 0.001), and between marriage residences (two-way ANOVA, *F*_1,612_ = 6.921, *p* < 0.01). (*b*) Similarly, mean age at first birth of females was significantly different among age cohort (two-way ANOVA, *F*_3,823_ = 14.435, *p* < 0.001), and between marriage residences (two-way ANOVA, *F*_1,823_ = 13.897, *p* < 0.001). There was no significant interaction between these two factors for either males or females. Unfilled bars, duolocal; filled bars, neolocal.

Neolocal residence accounted for 31 per cent of males and 27 per cent of females (330 males and 386 females). Neolocal couples gain access to their own share of the household land, by agreement or by making claim to the government, and this usually is associated with a new business opportunity, such as building a tourist hotel or other non-farming business [24]. Some neolocal households developed into group households, as the second generation did not disperse and resumed the duolocal system. We found neolocal Mosuo reproduced significantly earlier than duolocal Mosuo, among both males and females (figure 3), suggesting duolocal households are suffering resource constraints.

While it is possible that several relationships may have occurred before the first birth or after the last birth that our data do not reveal, children were reported by the maternal household to be from the same father for 97 per cent of mothers (*n* = 562). This corresponded with reports from the fathers' households (if they lived in the study area), in all but three individual cases. So formal fatherhood now seems to be largely agreed, but this may not always correspond to genetic paternity. It is possible that rates of polygyny and polyandry were higher in the past.

### (b) Relatedness

**Figure 4.**
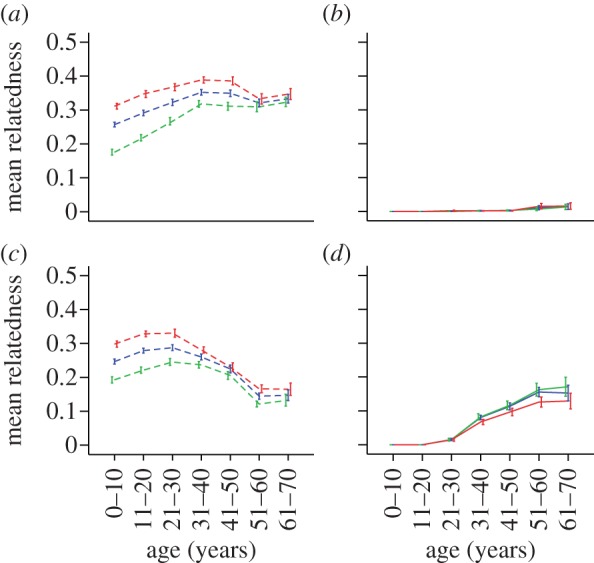
Age-specific relatedness to natal household and spouse's household for duolocal males and females (up to the age of 70). The averaged relatedness to all other females (red line), to all other males (green line) and to all other people of both sexes in the household (blue line) is plotted against age (cohort); (*a*) the averaged relatedness of a visiting marriage female to her natal household; (*b*) the averaged relatedness of a visiting marriage female to her spouse's household; (*c*) the averaged relatedness of a visiting marriage male to his natal household; and (*d*) the averaged relatedness of a visiting marriage male to his spouse's household. Error bars indicate mean±s.e.

Figure 4 shows how relatedness to the household varies with age in duolocal households for 750 males and 751 females living in households following the visiting marriage system. Females in a duolocal household are very closely related to each other throughout life (figure 4*a*), perhaps more highly related than in any other human social system. Females start life slightly less related to the males than to the females in the household, as their mothers are co-resident but their fathers are not; but their relatedness to the males increases throughout life (as uncles and cousins are replaced by brothers, nephews and sons over time). Males are closely related to females in their natal household at birth (figure 4*c*) but average relatedness to their household declines slowly with age (as mother and sisters are replaced by nephews and nieces over time); meanwhile their relatedness to their spouse's household is low but slowly increases with age as their children are born and their daughters reproduce themselves (figure 4*d*). Our data show that neolocal males tend to be older than those in other forms of marital residence (average age in a neolocal males was 52.2 ± 13.6, in duolocal marriage males was 42.2 ± 13.1); some reported that they had switched to living with their wife later in the life of a marriage that started as a visiting marriage. These results are consistent with the view that males are residing in the household to which they are most closely related on average and, for most of his life, a man is more closely related to his natal household than his spouse's household.

### (c) Labour patterns

**Figure 5.**
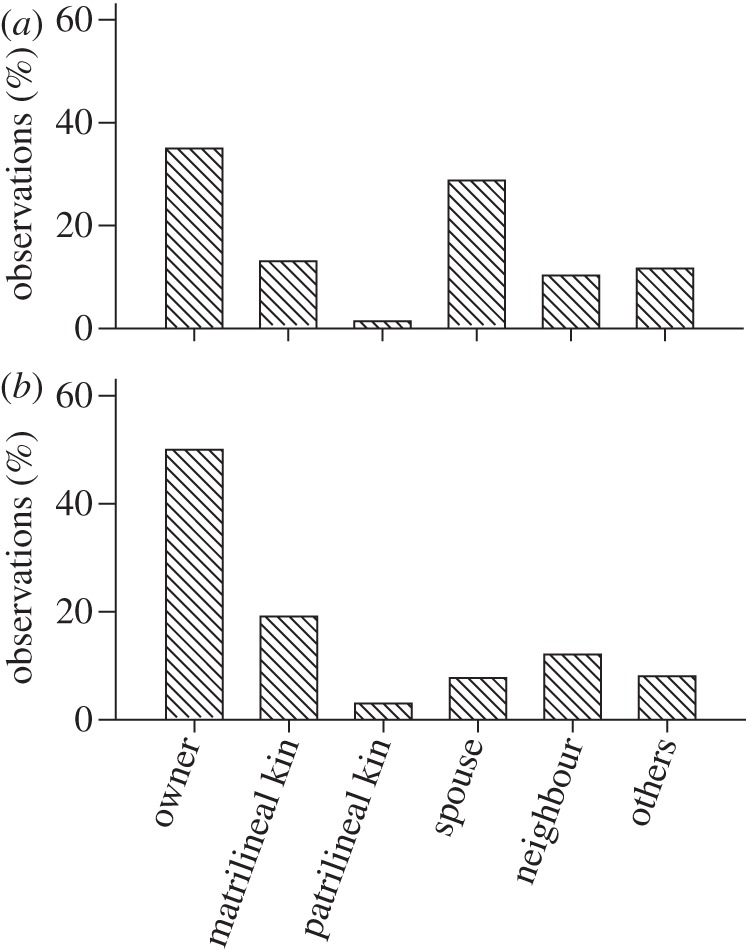
Relative allocation of labour during the planting season (% of observations taken from observing all those working on 159 farms, among farms of natal household, kin, spouse, neighbours and others by (*a*) duolocal Mosuo males (*n* = 146) and (*b*) duolocal Mosuo females (*n* = 298).

Using our spot observations over the planting season, we found that duolocal females are seen working more often on farms of their natal household and of matrilineal kin than are duolocal males; but they are seen less often working on farms of spouses than are duolocal males (figure 5, *χ*^2^_5_ = 39.46, *p* < 0.001).

A few Han, who are patrilineal and generally live in neolocal and nuclear families, also farm in the study area. Married Mosuo males were less likely to be seen working in the fields than were married Han males and were also seen less than married Mosuo females (table 1).

**Table 1.** Results of binomial test and *χ*^2^ analysis of males and females observed working in the fields in the planting season by ethnicity and marriage status (*n* = 104 Han, *n* = 839 Mosuo). (Han are patrilineal and mostly live in nuclear households.)

	male	female	exact significance (two-tailed; Binomial test)	**χ**^2^	d.f.	*p*-value
married						
Mosuo	191	396	0	3.9024	1	0.048
Han	33	42	0.356			
single						
Mosuo	140	112	0.089	0.002	1	0.969
Han	16	13	0.711			

Thus, Mosuo males work to some extent on both their natal farm and their spouse's farm, but are more often seen on the former. According to our model, this should occur if two or more breeding-age sisters co-reside, so this division of labour is consistent with the matrilineal bias in male work effort predicted by the model, irrespective of whether females or males are mating polygamously.

## 5. Discussion

Our results suggest that males in this system do not invest in matrilineal kin owing to high paternity uncertainty, nor are they being forced to invest in matrilineal kin against their reproductive interests. The more sisters (or other matrilineal kin) breed communally, the more male inclusive fitness is favoured by him working on his natal farm. Within this duolocal communal breeding system, males live in the household to which they are most closely related, which is where they are most likely to be fed in return for relatively little work. Indeed the description of the area in the Mosuo Regional Tourist Office leaflet describes it as ‘a paradise for men’.

The shortage of married Mosuo males working in the fields relative to both females and to Han males, suggests that mothers and sisters are willing to feed their adult sons and brothers while making relatively low demands on their labour. A man's natal household will experience inclusive fitness benefits from his mating effort, which his wife's household will not, so they may be more willing to invest in behaviours (such as resting and growing, socializing, and political activity) that may enhance his mating effort (either within or extra-pair). We argue that males do not work much on their spouses farms because of the communal nature of farming and child-rearing within households: when the benefits of labour are shared among all those resident in the communal household, the fitness benefit of investment in the household where his children reside is diluted by all the unrelated members of his wife's extended family with whom she is co-resident and shares food. A male's relatedness to his natal household is higher than to his spouse's household, but it does decrease with age, so a male's inclusive fitness returns on investment in his natal family will decline through his life, which may be why we observe that duolocal males are on average younger.

Why female kin breed communally is not addressed in our model, but our finding that males and females in neolocal households start reproducing at younger ages than those in duolocal households suggests that limited availability of resources for new households is a possible explanation for low dispersal. Few opportunities for dispersal has been associated with communal breeding in other species [25,26]. Furthermore, when the opportunity arises to invest exclusively in their own offspring by establishing neolocal nuclear households funded by tourist-related income, then the duolocal residence and visiting marriage system breaks down [24].

There are other examples in the region of communal households being associated with limited resources, notably the fraternally polyandrous Tibetan (Sherpa) communities of northwestern Nepal, who are patrilineal. There the farmland is constrained to a few river valleys surrounded by barren mountain slopes in the Himalayas, suggesting the habitat is saturated; in this case the household and farmland is inherited communally by brothers who marry one wife. This system also breaks down when new economic opportunities enable neolocal nuclear households to be established by younger brothers [27]. The Tibetans who exhibit this system live at high altitudes and may have adopted fraternal polyandry in preference to the duolocal system of the Mosuo owing to higher labour demands in that harsh habitat, requiring investment from several men to enable the reproductive success of one woman. Several European and Asian farming societies also face the problem of how to avoid dividing a farm between too many offspring. Other solutions include unigeniture (either primogeniture or ultimogeniture); but such systems normally arise where there are alternative income-earning opportunities for those offspring that fail to inherit land, such as in trade, the army or religious institutions. Tibet and the Tibetan border areas did not necessarily provide much in terms of alternative livelihoods (often only able to offer monastic celibacy to unmarried individuals). Patrilineal joint families were common elsewhere in pre-revolutionary China, where brothers and their spouses co-resided, but these households were inherently unstable as co-resident nuclear family units had separate interests and these joint families frequently dissolved into nuclear families on the death of the grandfather [1]. Much of the diversity in human marriage systems is now being lost as nuclear families become the norm globally in the face of economic development; although arguably new systems emerge all the time, for example, a decline in marital residence, low paternal investment and difficulty in affording housing causing multiple generations to co-reside are not uncommon phenomena in some urban settings [28].

In the Mosuo, and other duolocal systems, communal breeding by matrilineal kin is explicit in that there is a communal residence and communally owned farmland and communal cooking, eating and child-care; but it is interesting to speculate whether communal breeding by matrilineal kin is a more general explanation for males biasing their investment towards their natal family in matrilineal groups where breeding-age related females do not reside in the same dwelling. It is notable that elements of communal living are very common in many matrilineal groups. It has even been suggested that large houses are indicative of matrilineal kinship in archaeological sites, possibly suggestive of many relatives co-residing [29]. Some element of duolocality is sometimes observed in the early part of a marriage, with daughters then moving out after two or so births [30]. Males usually do not disperse far in matrilineal systems and remain in close contact with their mothers and sisters even if they do not reside with them, so all of the sibships are usually nearby. Communal farming in matrilineal work groups, often containing sisters, is common, as is the sharing of food, especially at times of need; sisters may be obligated to share the harvest [31]. And finally adoption of each other's children within the matriline is often common practice. All these elements of communal production and reproduction by female kin are almost indicative of matrilineal kinship systems and could favour male investment in his natal family farm for similar reasons to why it is favoured in duolocal Mosuo households.

By considering the fitness costs and benefits to individuals, we explain this rare marriage system, where husband and wife live apart, in terms of enhancing inclusive fitness in a habitat where resources used for breeding are shared by the entire household. In contrast to what has sometimes been assumed about human matrilineal systems, here we suggest that paternity uncertainty is not a cause of matriliny. Monogamy may even be favoured in some cases owing to its association with increasing relatedness in a communal household, as is the case in other cooperatively breeding species [32,33]. High levels of polygamous mating could reduce overall male work rates, but makes only a small contribution towards a male working on his sister's farm. It is the communal breeding of related females that is promoting matrilineal investment, duolocal residence and the visiting marriage.
